# Tumour enhancement with newly developed Mn-metalloporphyrin (HOP-9P) in magnetic resonance imaging of mice

**DOI:** 10.1054/bjoc.2001.1802

**Published:** 2001-06

**Authors:** Y Takehara, H Sakahara, H Masunaga, S Isogai, N Kodaira, H Takeda, T Saga, S Nakajima, I Sakata

**Affiliations:** Department of Radiology, Hamamatsu University School of Medicine, 3600 Handa, Hamamtsu 431-3192, Japan; Kyoto University Graduate School of Medicine; Health Care Center, Obihiro University of Agriculture and Veterinary Medicine; Photochemical Co. Ltd, Okayama, Japan

**Keywords:** animal, metalloporphyrins, manganese, contrast media, neoplasms, magnetic resonance imaging

## Abstract

The purpose of the study is to evaluate the tumour enhancing characteristics and biodistribution of a newly developed metalloporphyrin derivative, HOP-9P (13, 17-bis (1-carboxypropionyl) carbamoylethyl-3, 8-bis (1-phenylpropyloxyethyl)-2,7,12,18-tetra- methyl-porphynato manganese (III)). Seven mice bearing SCC VII tumours were imaged using T1-weighted conventional spin echo magnetic resonance images before and 5 min, 2 h and 24 h after intravenous injection of 0.1 mmol/kg of HOP-9P. For the acquired images, signal intensities of the tumour, muscle and oil-phantom were measured. Then, tumor/oil and tumor/muscle signal intensity ratios were calculated. Nineteen mice were sacrificed before or after the administration of HOP-9P (at 5 min, 2 h and 24 h), and the biodistribution of manganese in the tumour, muscle, liver, blood and kidneys was measured using optical emission spectrometers and was expressed as micrograms of manganese per gram of tissue. The tumour/muscle signal intensity ratio at 24 h (3.18 ± 0.34) was significantly higher than precontrast ratio (1.77 ± 0.20) (P < 0.05). The biodistribution assessment of manganese demonstrated that HOP-9P gradually and consistently accumulated in the tumour to reach the highest concentration at 24 h (3.49 ± 1.22 μ gMn/g). It is concluded that HOP-9P is a potential tumour-specific MR contrast agent. © 2001 Cancer Research Campaign http://www.bjcancer.com


*British Journal of Cancer* (2001) **84**(12), 1681–1685
© 2001 Cancer Research Campaign
doi: 10.1054/ bjoc.2001.1802, available online at http://www.idealibrary.com on IDEAL®

http://www.bjcancer.com

### Tumour enhancement with newly developed Mn-metalloporphyrin (HOP-9P) in magnetic resonance imaging of mice

**Y Takehara[1], H Sakahara[1], H Masunaga[1], S Isogai[1], N Kodaira[1], H Takeda[1], T Saga[2], S Nakajima[3] and I Sakata[4]**

[1]Department of Radiology, Hamamatsu University School of Medicine, 3600 Handa, Hamamtsu 431-3192, Japan; [2]Kyoto University Graduate School of Medicine; [3]Health Care Center, Obihiro University of Agriculture and Veterinary Medicine; [4]Photochemical Co. Ltd, Okayama, Japan

**Summary** The purpose of the study is to evaluate the tumour enhancing characteristics and biodistribution of a newly developed metalloporphyrin derivative, HOP-9P (13, 17-bis (1-carboxypropionyl) carbamoylethyl-3, 8-bis (1-phenylpropyloxyethyl)-2,7,12,18-tetra-methyl-porphynato manganese (III)). Seven mice bearing SCC VII tumours were imaged using T1-weighted conventional spin echo magnetic resonance images before and 5 min, 2 h and 24 h after intravenous injection of 0.1 mmol/kg of HOP-9P. For the acquired images, signal intensities of the tumour, muscle and oil-phantom were measured. Then, tumor/oil and tumor/muscle signal intensity ratios were calculated. Nineteen mice were sacrificed before or after the administration of HOP-9P (at 5 min, 2 h and 24 h), and the biodistribution of manganese in the tumour, muscle, liver, blood and kidneys was measured using optical emission spectrometers and was expressed as micrograms of manganese per gram of tissue. The tumour/muscle signal intensity ratio at 24 h (3.18 ± 0.34) was significantly higher than precontrast ratio (1.77 ± 0.20) ($P < 0.05$). The biodistribution assessment of manganese demonstrated that HOP-9P gradually and consistently accumulated in the tumour to reach the highest concentration at 24 h (3.49 ± 1.22 μ gMn/g). It is concluded that HOP-9P is a potential tumour-specific MR contrast agent. © 2001 Cancer Research Campaign http://www.bjcancer.com

**Keywords**: animal; metalloporphyrins; manganese; contrast media; neoplasms; magnetic resonance imaging

Discriminating between tumours and normal tissues non-invasively is one of the major goals of diagnostic MR imaging. Although tumours are often hypointense on T1-weighted images and hyperintense on T2-weighted images compared to normal tissues even without any contrast media, these findings are not consistently specific. Therefore, a tumour-specific MRI contrast agent remains important for improved detection of tumours, staging, and their characterization. Gadolinium chelates are now being used as the workhorse for detecting tumours in the clinical environment. These contrast media, however, are non-specific extravascular contrast agents. Gadolinium chelates are rapidly delivered to tumours depending on their vascular space, and stay in the tumour depending on the size of the extracellular compartment; however in the majority of the cases, they are rapidly washed out of the tumour. Therefore, the imaging windows for tumour depiction using gadolinium chelates have been quite narrow, and furthermore, the detection of hypovascular tumours has always been problematic with non-specific agents.

Porphyrins were the first potentially tissue-specific MR contrast agents to be evaluated because of their ability to form stable chelate complexes with paramagnetic metal ions and their selective retention by tumours. Moreover, complexes containing manganese were identified as potentially the most useful ones because of the high relaxivity of this metal. 2, 4-bis (1-tetrahydro-fulfuroxyethyl)-deuteroporphynyl (IX)-6-7-bisaspartic acid (THF-Mn-ASP), a prototype of HOP-9P, has shown its tumour specific characteristics in a previous report (Suzuki et al, 1996). In this study we perform an initial evaluation of the recently developed HOP-9P as a new tumour seeking MR contrast media using mice in comparison to conventional gadolinium chelates.

*Received 7 September 2000*
*Revised 5 March 2001*
*Accepted 6 March 2001*

*Correspondence to:* Y Takehara

### MATERIALS AND METHODS

### Chemicals

The compound, 13, 17-bis (1-carboxypropionyl) carbamoylethyl-3, 8-bis (1-phenylpropyloxyethyl)-2, 7, 12, 18-tetramethyl-porphynato manganese (III) (HOP-9P) has a molecular weight of 1135. In this study, HOP-9P was dissolved with 0.1 M phosphate buffer (pH8.0), and gadolinium-diethylenetriaminepentaacetic acid (Gd-DTPA) (Magnevist, Nihon Schering, Co. Ltd) was diluted with physiological saline before use.

### Phantom study for relaxivity measurements

The relaxivity of HOP-9P was measured at 37˚C in 0.9% sodium chloride solution in a 50 ml polypropylene centrifuge tube using a 1.5T super-conductive imager (Magnetom SP, Siemens Medical Systems, Erlangen, Germany). Relaxivity of Gd-DTPA was also measured for comparison. The T1 relaxation times for each solutions were calculated using the spin echo sequence (SE) (TR of 400 and 2400 ms), with 15 ms of TE. A matrix of 256 × 256 and slice thickness of 8 mm were used for the T1 measurements. Five different concentrations 1 M, 0.5 M, 0.25 M, 0.125 M, and 0.0625 M were prepared both for HOP-9P and Gd-DTPA solution for relaxivity plotting.

### Animal study

### *Tumour model*

SCC VII squamous cell carcinomas derived from C3H mice are maintained in Eagle's minimum essential medium. Approximately $1.0 \times 10^6$ cells were inoculated subcutaneously into the right flank of 6-week-old male C3H/He mice (SLC., Co. Ltd Shizuoka, Japan). Fourteen days after the inoculation, mice bearing tumour with 1–3 cm in diameter were used in this experiment. All procedures involving animals were carried out in accordance with the regulations for animal welfare in the university.

### *In vivo MR imaging*

Seven mice were used for HOP-9P enhanced MR imaging, and five mice were used for Gd-DTPA enhanced imaging. 1.5T superconductive imager (Signa Horizon Echospeed, GE Medical systems, WI, USA) together with a 5-inch surface coil was used for imaging. Under general anesthesia induced by an intraperitoneal injection of pentobarbital (30–50 mg/kg), T1-weighted conventional images and T2-weighted fast spin echo images were acquired before contrast administration. T1-weighted spin echo imaging was repeated 5 min, 2 h and 24 h after intravenous injection of HOP-9P, and 5 min, 30 min, 1 h and 2 h after administration of Gd-DTPA. Parameters used for T1 weighted imaging were TR (ms)/TE (msec) of 500/35, a field of view (FOV) (cm) of $12 \times 12$, NEX of 1, image matrix of $256 \times 128$, and slice thickness of 3 mm. Coronal interleave scan was performed in 2 min and 24 s. For coronal T2 weighted imaging, TR (ms)/TE (ms) of 2000/90, echo train length of 8, FOV (cm) of $12 \times 12$, NEX of 1, image matrix of $256 \times 128$, slice thickness of 3 mm and slice gap of 3 mm were used. The imaging time for the T2 weighted imaging was one minute and 12 s. 0.1 mmol/kg (0.01 ml/g) of either of HOP-9P or Gd-DTPA was injected into the lateral tail vein of the mice. A water phantom and an oil phantom were also included in the FOV.

### *Quantitative image analysis*

On a network computer, mean signal intensities were measured by focusing circular regions of interest on the tumour, muscle and oil phantom. Tumour/oil signal intensity ratio (SIR) and tumor/muscle SIR were then calculated.

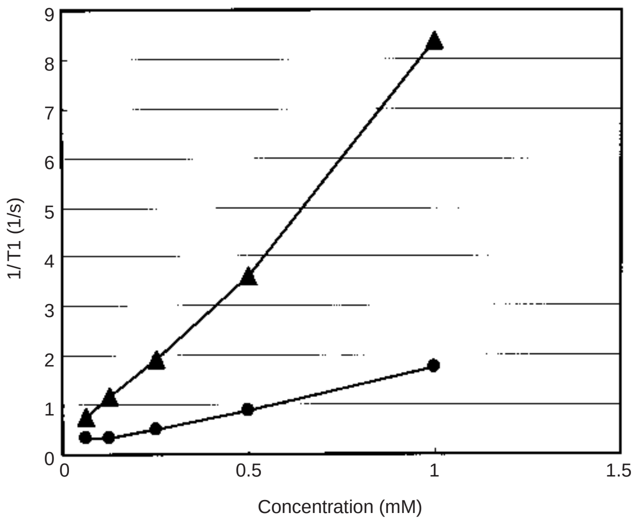

**Figure 1** Spin lattice relaxation rates (1/T1) of water at 1.5T as a function of concentration (M) of two contrast media; Gd-DTPA (line with circular marks) and HOP-9P (line with triangular marks). The relaxivity of HOP-9P is about four-fold compared to that of Gd-DTPA

### *Biodistribution analysis*

Nineteen mice were sacrificed by cervical dislocation 5 min, 2 h and 24 h after the administration of HOP-9P. Eleven mice were sacrificed in the same manner after Gd-DTPA injection. In addition, eight mice were used for control study. Manganese and gadolinium concentrations in the blood, tumor, muscle, liver and kidneys were quantified using optical emission spectrometers (SPS 1200A, Seiko Instruments Inc., Chiba, Japan) with inductively coupled argon plasma (ICAP), and were expressed as μg of manganese or gadolinium per gram of tissue. The wavelengths used for measurements were 342.247 nm for gadolinium and 257.610 nm for manganese.

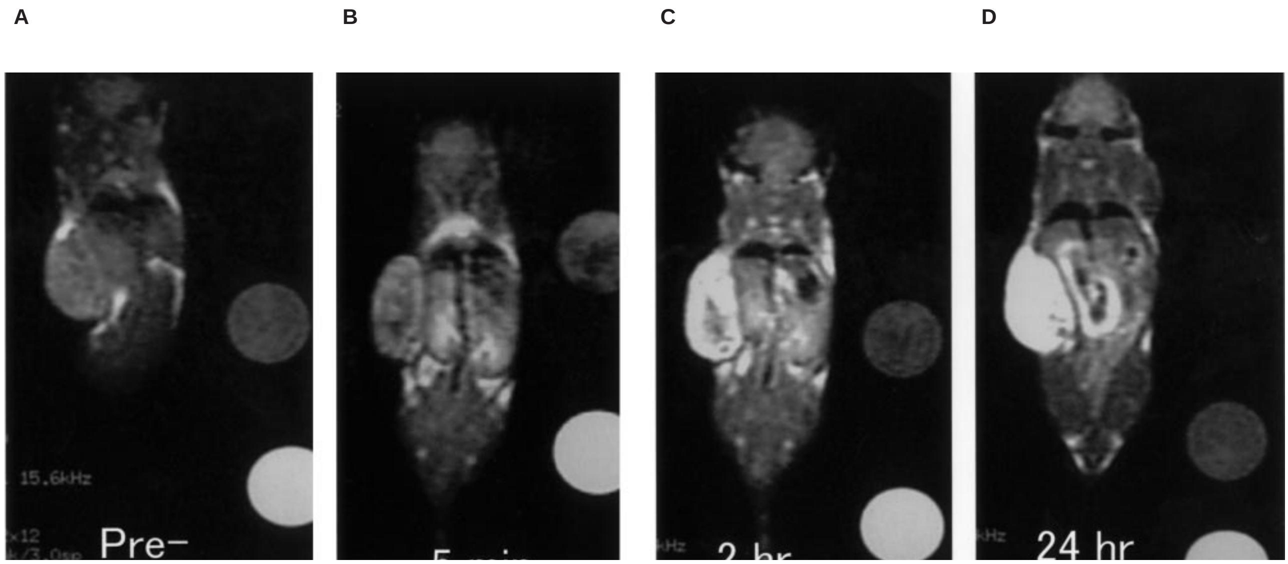

**Figure 2** Representative MR images acquired pre- and post-HOP-9P injection (0.1 mmol/kg). Repeated acquisitions of T1 weighted images with use of spin echo sequence (TR/TE: 500/35). Precontrast (**A**), 5 min (**B**), 2 h (**C**) and 24 h (**D**) after contrast injection. Note progressive and marked enhancement of the tumour in the right flank

*© 2001 Cancer Research Campaign*

### Statistical analysis

Data are expressed as means ± SD. Differences between SI values obtained before and after administration of contrast material within each group were evaluated with a repeated-measure analysis of variance followed by the Fisher's PLSD test. Differences of metal concentrations between pre-enhancement and post-enhancement for tumour and other organs were analysed using a Student's *t*-test, where Bartlett's test indicated homogeneity of variance, or by a non-parametric Mann-Whitney test. $P < 0.05$ was accepted as statistically significant.

### RESULTS

A linear correlation between the concentration of the paramagnetic agents and longitudinal relaxivity was found. The relaxivity of HOP-9P was consistently stronger than Gd-DTPA (Figure 1).

MR images demonstrated significant enhancement of transplanted tumour after intravenous injection of HOP-9P (Figure 2). Time course changes in ratio of SI of tumour/oil and tumour/muscle are shown in Table 1. The tumour/oil SIR peaked at 24 h (0.75 ± 0.17) compared to precontrast ratio (0.36 ± 0.03) ($P < 0.05$). The tumour/muscle SIR was highest at 24 h (3.18 ± 0.34) compared to precontrast ratio (1.77 ± 0.20) ($P < 0.05$). Mean percent increase in signal intensity at 24 h was 124% in the tumour/oil ratio and 79% in tumour/muscle ratio. The concentration of manganese after 0.1 mmol/kg HOP-9P injection in the blood was 24.7 ± 2.47 μ gMn/g tissue at 5 min. HOP-9P rapidly distributed to the liver and kidneys, and afterward, it was gradually washed out of or excreted from the organs. Tumours showed gradual and selective retention of the metal complex with the highest value of 3.49 ± 1.22 μg Mn/g reached at 24 h following intravenous administration (Table 1). The metal concentration in tumors remained consistently higher than muscle both at 2 h and 24 h and a three- to four-fold increase in tumour/muscle concentration ratio over the control was seen at 24 h.

Tumour enhancement was recognized in mice receiving Gd-DTPA at 5 min after contrast injection, however it faded out thereafter (Figure 3). Tumour conspicuity on the images increased by 30% in tumour/oil ratio and 19% in tumour/muscle ratio, at 5 min and then decreased. Gadolinium concentration in the tumor was similar to that in the bloodstream with the highest concentration of 10.3 ± 1.74 μ gGd/g at 5 min after contrast injection. Two hours after Gd-DTPA injection, gadolinium was virtually washed out from the tumor with a tissue concentration of 0.38 ± 0.28 μg Gd/g (Table 1).

### DISCUSSION

Since an initial investigation by Chen et al, metalloporphyrin derivatives using manganese have been studied by several groups and shown to significantly enhance the conspicuity of a number of murine tumours and human tumour xenografts in murine hosts (Chen et al, 1984). These studies were generally undertaken with the objective of developing tumour-specific paramagnetic contrast enhancing agents. Enhancement of the MRI signal from tumour has been known to correlate with a high concentration of metalloporphyrins in tumor tissue. The uptake of porphyrins by tumours is not fully understood, but may include selective transport and

**A** **B** **C**

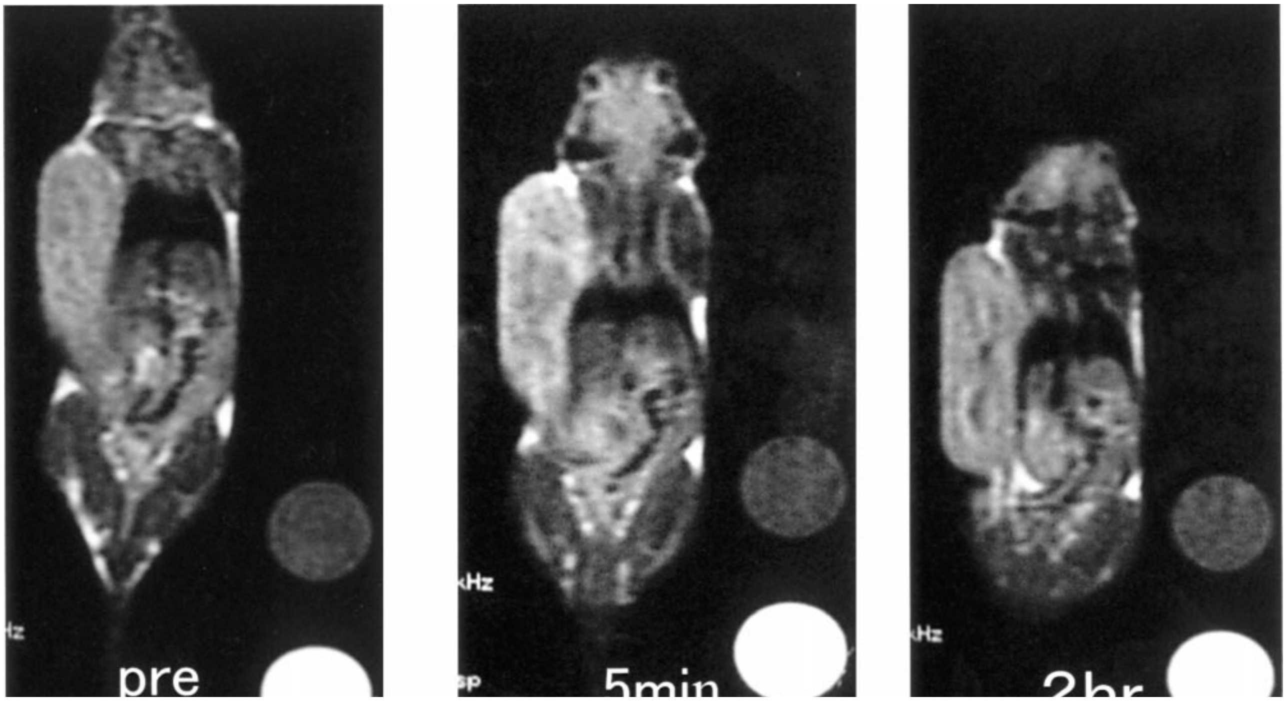

**Figure 3** Representative MR images obtained before (**A**) and after (**B** and **C**) intravenous administration of Gd-DTPA (0.1 mmol/kg). The tumour in the right flank is moderately enhanced 5 min after the injection. The lesion conspicuity after contrast injection, however, is less than that acquired with HOP-9P. The tumour enhancement faded out 2 h after injection (**C**).

© 2001 Cancer Research Campaign 

**Table 1** Signal intensity ratio measured by imaging studies and metal concentration values measured by biodistribution analysis

| Time | Tumour/oil SIR (mean +/–SD) | | Tumour/muscle SIR (mean +/–SD) | | Metal conc. in Tumour ($\mu$g/g) (mean +/–SD) | |
|---|---|---|---|---|---|---|
| | Gd-DTPA | HOP-9P | Gd-DTPA | HOP-9P | Gd-DTPA | HOP-9P |
| Pre-contrast | 0.38+/–0.03 | 0.36+/–0.03 | 1.68+/–0.12 | 1.7+/–0.2 | 0 | 0.26+/–0.11 |
| 5 min | 0.50+/–0.11* | 0.46+/–0.04* | 1.98+/–0.41 | 1.74+/–0.18 | 10.3+/–1.74 | 1.15+/–0.30* |
| 30 min | 0.47+/–0.08* | NA | 1.88+/–0.17 | NA | NA | NA |
| 1 h | 0.47+/–0.09* | NA | 1.80+/–0.15 | NA | NA | NA |
| 2 h | 0.44+/–0.08 | 0.63+/–0.11* | 1.75+/–0.11 | 2.05+/–0.19* | 0.38+/–0.28 | 2.87+/–0.86* |
| 24 h | NA | 0.75+/–0.17* | NA | 3.18+/–0.34* | 0.19+/–0.01 | 3.49+/–1.22* |

SIR: signal intensity ratio; Conc: concentration; NA: not applicable (not measured); *significant increase compared with pre-contrast value ($P < 0.05$).

passive diffusion. It is said that hydrophilic agents distribute into extracellular stroma, whereas hydrophobic compounds show intracellular distribution (Megnin et al, 1987; Oenbrink et al, 1988). Several hypotheses have been proposed concerning this high concentration of metalloporphyrins in the tumour. First, a combination of high capillary permeability and the lack of lymphatic drainage in the neoplasm may result in the stasis of porphyrin. Second, porphyrins have strong affinities to stromal elements such as highly concentrated collagenous tissues found in the large interstitial space of tumors (Jain, 1987; Kessel et al, 1983). Third, some metalloporphyrin derivatives have high affinity for low-density lipoprotein (LDL), and the uptake is dependent on the lipophilic feature of them. It is generally accepted that in neoplastic cells, LDL receptor distribution is markedly increased (Dagan et al, 1995; Niendorf et al, 1995). Lipophilic porphyrins may be taken into the cancer cells via LDL receptor pathways (Fiel et al, 1988; Kessel et al, 1983; Nakajima et al, 1995; Reyftmann et al, 1984; Takemura et al, 1994). Lastly, some of the water-soluble porphyrins may be bound to a β-glycoprotein such as haemopexin that is structurally similar to hyaluronidase (Nakajima et al, 2000; Zhu et al, 1994). Hyaluronidase is an enzyme involved in tumour proliferation and metastasis as well as in neovascularization. Some of the water-soluble porphyrins may be carried into tumours by haemopexin (Nakajima et al, 2000).

This study has revealed that HOP-9P, similar to other Mn-porphyrins, causes effective and potent shortening of T1 value not only in vitro (a four-fold relaxivity compared to Gd-DTPA), but also in vivo. Koenig et al had previously discussed the mechanism of this unusually high relaxivity of Mn-porphyrins. They suggested the effect may be mainly due to the anisotropy of the ground-state wave function of $Mn^{3+}$ in the porphyrin complex, effectively bringing the spin density of the $Mn^{3+}$ ions closer to the protons of the coordinated water molecules (Koenig et al, 1987). This effect is favourable for use as contrast agents, because a high relaxivity can reduce the dosage required to achieve a given enhancement of contrast in MRI, thereby reducing the risk of toxicity (Koenig et al, 1987).

Mn-porphyrin HOP-9P was distributed in the circulation system rapidly during the first 5 min and therefore, the concentration of HOP-9P in the muscle was initially high. This resulted in low tumour/muscle contrast shown on the images at 5 min. However, HOP-9P was rapidly removed from the muscle likely due to back diffusion into the circulation system, and accumulated by the tumour gradually and consistently. This resulted in an increase in contrast between the tumour and muscle on the MR imaging at 2 h and 24 h. Tumour enhancement was much improved by HOP-9P in comparison to the prototype THF-Mn-ASP. The maximum increase of tumour/muscle intensity ratio was 32% using THF-Mn-ASP (Suzuki et al, 1996), whereas a 79% increase was observed in this study with HOP-9P with the same dose in mice.

As a tool for diagnostic imaging strategy, HOP-9P has unique characteristics for tumour detection. Unlike non-specific extravascular contrast media such as Gd-DTPA, the imaging window for detection of neoplasm with HOP-9P is much wider. In this study, we employed conventional spin echo sequence and normal contrast injection strategies in acquiring enhanced T1 weighted images, which required 2 min and 24 s. Based on the clinical experiences, however, optimum-imaging timing after Gd- DTPA injection is the first pass of the contrast, which is within 1 min. Yet, in our study, it was not practical to accomplish bolus injection of the contrast media inside the magnet. Typically, in the clinical setting, combined use of fast MR imager equipped with high performance gradient system and a power injector for bolus injection of the contrast media are necessary. Unlike Gd-DTPA, HOP-9P may not require the state-of-the-art equipment and strategies. A conventional MR system with lower Tesla magnet with ordinary contrast infusion will likely enable sufficient tumour discriminations.

Aside from diagnostic usage, porphyrin derivatives have also been developed as an agent for photodynamic therapy (Calzavara-Pinton et al, 1996; Hill et al, 1996; Kessel, 1992; Kostenich et al, 1997; Lapes et al, 1996; McIlroy et al, 1998; Nakajima et al, 1998; Shopova et al, 1994; Takemura et al, 1992; Woodburn et al, 1998), radiation sensitizer (Rowinsky 1999; Viala et al, 1999) and an agent for neutron capture therapy (Matsumura et al, 1999; Miura et al, 1996; Shibata et al, 1998). This study revealed that the enhancement effect of the tumour demonstrated on the MR images was well correlated with the biodistribution of the HOP-9P (Figure 2, Table 1). MR imaging using HOP-9P might be a safe and useful tool for predicting the effectiveness of the photodynamic therapy and radiation therapy including neutron capture. It may also be used to evaluate the outcome of those therapeutic procedures.

THF-Mn-ASP, a prototype of HOP-9P, has an LD50 of more than 2000 mg/kg in mice (Kobayashi et al, 1999), and HOP-9P has no known phototoxicity. Given these characteristics, HOP-9P may be a safe compound for MR imaging.

Although a detailed study of the pharmacokinetics and toxicology of this agent is needed before its usefulness as tumour-specific contrast media is fully established, current biodistribution analysis and the imaging study have suggested that HOP-9P is a potentially tumour specific contrast media in an animal model.

### REFERENCES

Calzavara-Pinton PG, Szeimies RM, Ortel B and Zane C (1996) Photodynamic therapy with systemic administration of photosensitizers in dermatology. [Review] [35 refs]. *J Photochem Photobiol* **B 36**: 225–231

*© 2001 Cancer Research Campaign*

Chen CW, Cohen JS, Myers CE and Sohn M (1984) Paramagnetic metalloporphyrins as potential contrast agents in NMR imaging. *FEBS Lett* **168**: 70–74

Dagan A, Gatt S, Cerbu-Karabat S, Maziere JC, Maziere C, Santus R, Engelhardt EL, Yeh KA, Stobbe CC and Fenning MC (1995) Uptake by cells and photosensitizing effectiveness of novel pheophorbide derivatives in vitro. *Int J Cancer* **63**: 831–839

Fiel RJ, Mark E, Button T, Gilani S and Musser D (1988) Mechanism of the localization of manganese (III) mesotetra(4-sulfonatophenyl) porphine in mice bearing L1210 tumors. *Cancer Lett* **40**: 23–32

Hill RA, Garrett J, Reddi S, Esterowitz T, Liaw LH, Ryan J, Shirk J, Kenney M, Shimuzu S and Berns MW (1996) Photodynamic therapy (PDT) of the ciliary body with silicon naphthalocyanine (SINc) in rabbits. *Lasers Surg Med* **18**: 86–91

Jain RK (1987) Transport of molecules in the tumor interstitium: a review. [Review] [122 refs]. *Cancer Res* **47**: 3039–3051

Kessel D and Chou TH (1983) Tumor-localizing components of the porphyrin preparation hematoporphyrin derivative. *Cancer Res* **43**: 1994–1999

Kessel D (1992) Photodynamic therapy and neoplastic disease. [Review] [78 refs]. *Oncol Res* **4**: 219–225

Kobayashi M, Tajiri H, Hayashi T, Kuroki M and Sakata I (1999) Tumor-enhancement effect of a Mn3+ metalloporphyrin derivative (ATN-4T) in magnetic resonance imaging. *Cancer Lett* **137**: 83–89

Koenig SH, Brown RD and Spiller M (1987) The anomalous relaxivity of Mn3+ (TPPS4). *Magn Reson Med* **4**: 252–260

Kostenich G, Orenstein A, Roitman L, Malik Z and Ehrenberg B (1997) In vivo photodynamic therapy with the new near-IR absorbing water soluble photosensitizer lutetium texaphyrin and a high intensity pulsed light delivery system. *J Photochem Photobiol* **B 39**: 36–42

Lapes M, Petera J and Jirsa M (1996) Photodynamic therapy of cutaneous metastases of breast cancer after local application of meso-tetra-(para-sulphophenyl)-porphin (TPPS4). *J Photochem Photobiol* **B 36**: 205–207

Matsumura A, Shibata Y, Yamamoto T, Yoshida F, Isobe T, Nakai K, Hayakawa Y, Kiriya M, Shimojo N, Ono K, Sakata I, Nakajima S, Okumura M and Nose T (1999) A new boronated porphyrin (STA-BX909) for neutron capture therapy: an in vitro survival assay and in vivo tissue uptake study. *Cancer Lett* **141**: 203–209

McIlroy BW, Curnow A, Buonaccorsi G, Scott MA, Bown SG and MacRobert AJ (1998) Spatial measurement of oxygen levels during photodynamic therapy using time-resolved optical spectroscopy. *J Photochem Photobiol* **B 43**: 47–55

Megnin F, Faustino PJ, Lyon RC, Lelkes PI and Cohen JS (1987) Studies on the mechanism of selective retention of porphyrins and metalloporphyrins by cancer cells. *Biochim Biophys Acta* **929**: 173–181

Miura M, Micca PL, Fisher CD, Heinrichs JC, Donaldson JA, Finkel GC and Slatkin DN (1996) Synthesis of a nickel tetracarboranylphenylporphyrin for boron neutron-capture therapy: biodistribution and toxicity in tumor-bearing mice. *Int J Cancer* **68**: 114–119

Nakajima S, Takemura T and Sakata I (1995) Tumor-localizing activity of porphyrin and its affinity to LDL, transferrin. *Cancer Lett* **92**: 113–118

Nakajima S, Sakata I, Hirano T and Takemura T (1998) Therapeutic effect of interstitial photodynamic therapy using ATX-S10(Na) and a diode laser on radio-resistant SCCVII tumors of C3H/He mice. *Anticancer Drugs* **9**: 539–543

Nakajima S, Moriyama T, Hayashi H, Sakata I, Nakae Y and Takemura T (2000) Hemopexin as a carrier protein of tumor-localizing Ga-metalloporphyrin-ATN-2. *Cancer Lett* **149**: 221–226

Niendorf A, Nagele H, Gerding D, Meyer-Pannwitt U and Gebhardt A (1995) Increased LDL receptor mRNA expression in colon cancer is correlated with a rise in plasma cholesterol levels after curative surgery. *Int J Cancer* **61**: 461–464

Oenbrink G, Jurgenlimke P and Gabel D (1988) Accumulation of porphyrins in cells: influence of hydrophobicity aggregation and protein binding. *Photochem Photobiol* **48**: 451–456

Reyftmann JP, Morliere P, Goldstein S, Satus R, Dubertret L and Lagrange D (1984) Interaction of human serum low density lipoproteins with porphyrins: a spectroscopic and photochemical study. *Photochem Photobiol* **40**: 721–729

Rowinsky EK (1999) Novel radiation sensitizers targeting tissue hypoxia. [Review] [84 refs]. *Oncology (Huntingt)* **13**: 61–70

Shibata Y, Matsumura A, Yamamoto T, Nakagawa K, Yoshii Y, Nose T, Sakata I, Nakajima S, Hayakawa Y and Ono K (1998) Neutron capture therapy with a new boron-porphyrin compound in the rat 9L glioma model. *J Exp Clin Cancer Res* **17**: 285–289

Shopova M, Wohrle D, Stoichkova N, Milev A, Mantareva V, Muller S, Kassabov K and Georgiev K (1994) Hydrophobic Zn(II)-naphthalocyanines as photodynamic therapy agents for Lewis lung carcinoma. *J Photochem Photobiol* **B 23**: 35–42

Suzuki T, Nakano K, Tomiyoshi K, Sakata I, Endo K and Yamanaka H (1996) Contrast enhancement of PC-3 prostate cancer for magnetic resonance imaging: animal studies using tumor-localizing Mn-metalloporphyrin (THF-Mn-Asp) *J Urol* **156**: 1850–1852

Takemura T, Ohta N, Nakajima S and Sakata I (1992) The mechanism of photosensitization in photodynamic therapy: chemiluminescence caused by photosensitization of porphyrins in saline containing human serum albumin. *Photochem Photobiol* **55**: 137–140

Takemura T, Nakajima S and Sakata I (1994) Tumor-localizing fluorescent diagnostic agents without phototoxicity. *Photochem Photobiol* **59**: 366–370

Viala J, Vanel D, Meingan P, Lartigau E, Carde P and Renschler M (1999) Phases IB and II multidose trial of gadolinium texaphyrin, a radiation sensitizer detectable at MR imaging: preliminary results in brain metastases. *Radiology* **212**: 755–759

Woodburn KW, Fan Q, Kessel D, Luo Y and Young SW (1998) Photodynamic therapy of B16F10 murine melanoma with lutetium texaphyrin. *J Invest Dermatol* **110**: 746–751

Zhu L, Hope TJ, Hall J, Davies A, Stern M, Muller-Eberhard U, Stern R and Parslow TG (1994) Molecular cloning of a mammalian hyaluronidase reveals identity with hemopexin, a serum heme-binding protein. *J Biol Chem* **269**: 32092–32097

© 2001 Cancer Research Campaign
